# Bridging Generations for Health and Social Cohesion: A Community-Based Intergenerational Model From Rural India

**DOI:** 10.7759/cureus.104898

**Published:** 2026-03-09

**Authors:** Arjunkumar H Jakasania, Swati Misra, Subodh S Gupta

**Affiliations:** 1 Department of Community Medicine, Mahatma Gandhi Institute of Medical Sciences, Sevagram, Wardha, IND

**Keywords:** cascade training model, community engagement, healthy ageing, intergenerational programs, social connectedness

## Abstract

India’s rapidly aging population and the decline of joint family systems have increased social isolation among older adults while weakening intergenerational ties. This brief report describes *Sahjeevan-Chaitanya Natyanche *(Celebrating Generations), a community-based intergenerational program implemented across 18 villages (population ~31,000) in rural Maharashtra. Using a cascade training model, the program engaged key trainers, master trainers, and peer trainers to promote intergenerational dialogue, adolescent life skills, and elderly self-care. A mixed-method baseline-endline evaluation design was employed, including stratified random sampling and standardized tools such as the WHO-QOL BREF, Social Capital Scale, and Loyola Generativity Scale, complemented by qualitative methods. Findings suggested increases in elders’ self-perceived usefulness, social connectedness, and reported intergenerational engagement. The implementation experience highlights the feasibility of low-cost, locally adapted intergenerational programs in resource-limited rural settings. Integration of such approaches within existing community and primary healthcare systems warrants further systematic evaluation.

## Editorial

Introduction

Over the past few decades, demographic transition has reshaped the age distribution in countries worldwide. India stands at a demographic crossroads, with its elderly population expected to surge from 104 million today to nearly 319 million by 2050 [1]. As India’s population ages rapidly, changes like urbanization and migration are reducing contact between the younger and older generations. This has led to more older adults feeling lonely, socially isolated, and having a lower quality of life [2,3]. At the same time, the decline of traditional joint families has weakened community ties and interrupted the sharing of cultural values, social support, and caregiving between generations [4].

Against this backdrop, intergenerational programming (IGP) represents a culturally adaptable strategy that has been explored as an approach to addressing social isolation and strengthening intergenerational relationships. By creating opportunities for older adults and younger generations to interact and learn from each other, IGP helps reduce the emotional and social challenges of aging. At the same time, it supports the growth and development of children and adolescents [5,6]. The intergenerational program, *Sahjeevan-Chaitanya Natyanche* (Celebrating Generations) in Wardha district of Maharashtra, India, is a strong example of how such an approach can be adapted to local needs and successfully implemented on a larger scale, building on global experiences.

The growing challenge: aging and social isolation

In India, many older adults are facing growing challenges to their social well-being [7]. As more young people move to cities and nuclear families become the norm, the role of the elderly within homes and communities is shrinking. Negative attitudes toward aging also make older people feel less valued [7,8]. A few studies have shown that social isolation among the elderly can lead to problems like depression, memory loss, and poor self-care [2-4]. This demographic transition has implications not only for population structure but also for psychosocial well-being. Addressing it requires more than just medical solutions; we need approaches that rebuild connections and community support.

The promise of intergenerational engagement

Internationally, intergenerational models have been recognized for enhancing elder well-being and youth development [9]. In countries like the United States, the United Kingdom, and Japan, such programs bring the elderly and children together in schools, day-care centers, and community spaces [10-12]. These interactions improve elders’ sense of purpose and reduce loneliness, while children benefit from mentorship, cultural knowledge, and emotional support. However, much of this evidence comes from high-income countries, with limited data from low- and middle-income contexts such as India. Furthermore, existing models are often facility-based and exclude children aged 5-10, a vital age for cognitive and emotional development.

Sahjeevan: a locally rooted model of IGP

**Figure 1:**
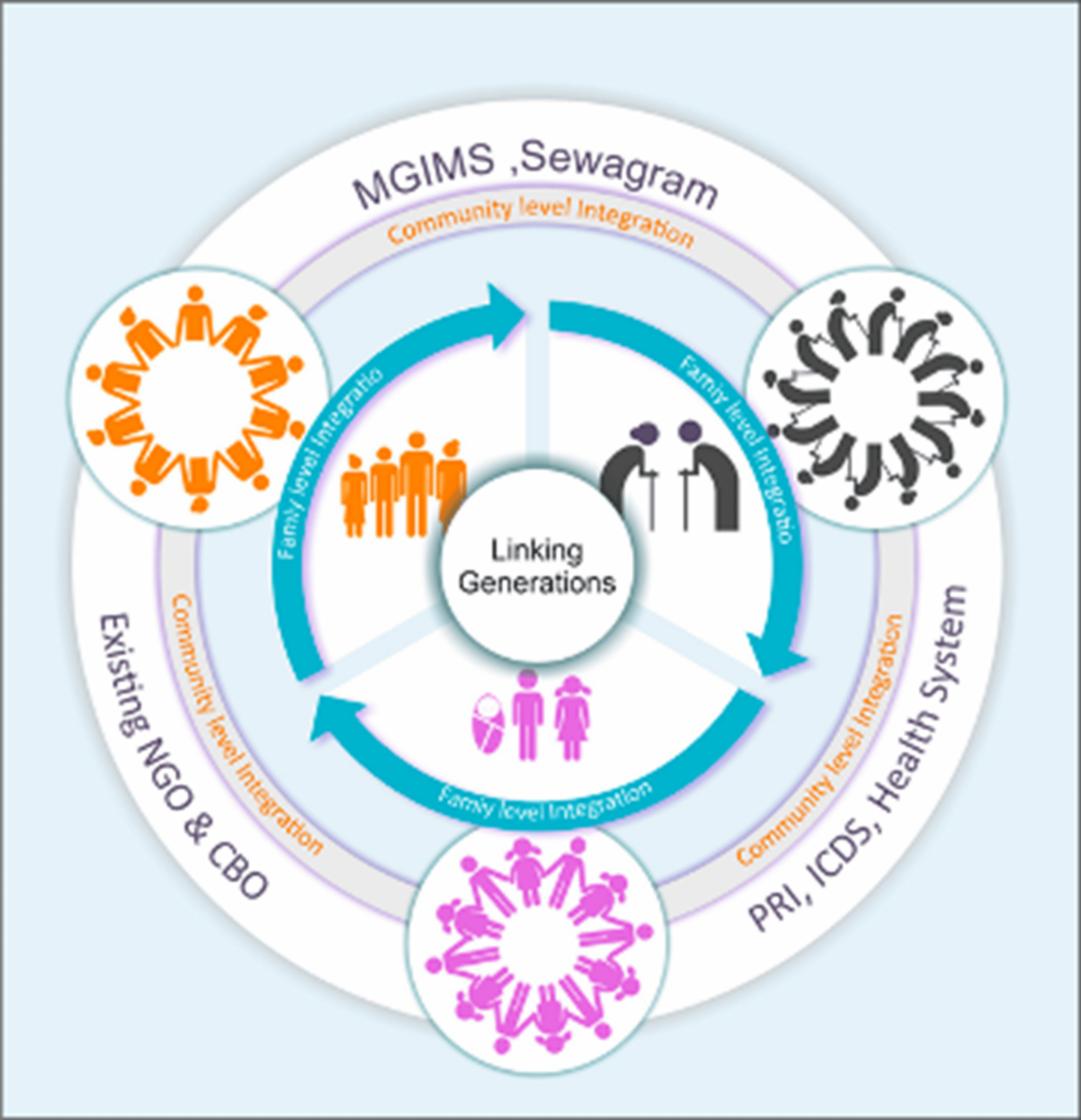
Community-based model for linking generations through family and system-level integration Source: Developed by the authors using Microsoft PowerPoint (Microsoft Corporation, Redmond, WA, US) as part of the intergenerational project implemented by the Mahatma Gandhi Institute of Medical Sciences, Sevagram.

The *Sahjeevan-Chaitanya Natyanche* project is implemented by the Mahatma Gandhi Institute of Medical Sciences (MGIMS), Sevagaram, Maharashtra, India, in partnership with the World Health Organization (WHO). The Department of Community Medicine (DCM) and MGIMS collaborated with *Adharwad* (local-level partnership), a non-government organization (NGO) in the Wardha district of Maharashtra (Figure 1). Frontline functionaries, such as *ASHA *(Accredited Social Health Activists) and *Anganwadi* workers, were involved in implementation at the village level. Gram panchayat-level assistance was also received in terms of resources such as place and chairs. The project is implemented in 18 villages in 5 PHC areas (Anji, Bhidi, Kharangna, Talegoan, and Waifad) in Wardha district, comprising approximately 31000 population.

Key features of the program included the following.

Cascade Training Model

**Figure 2:**
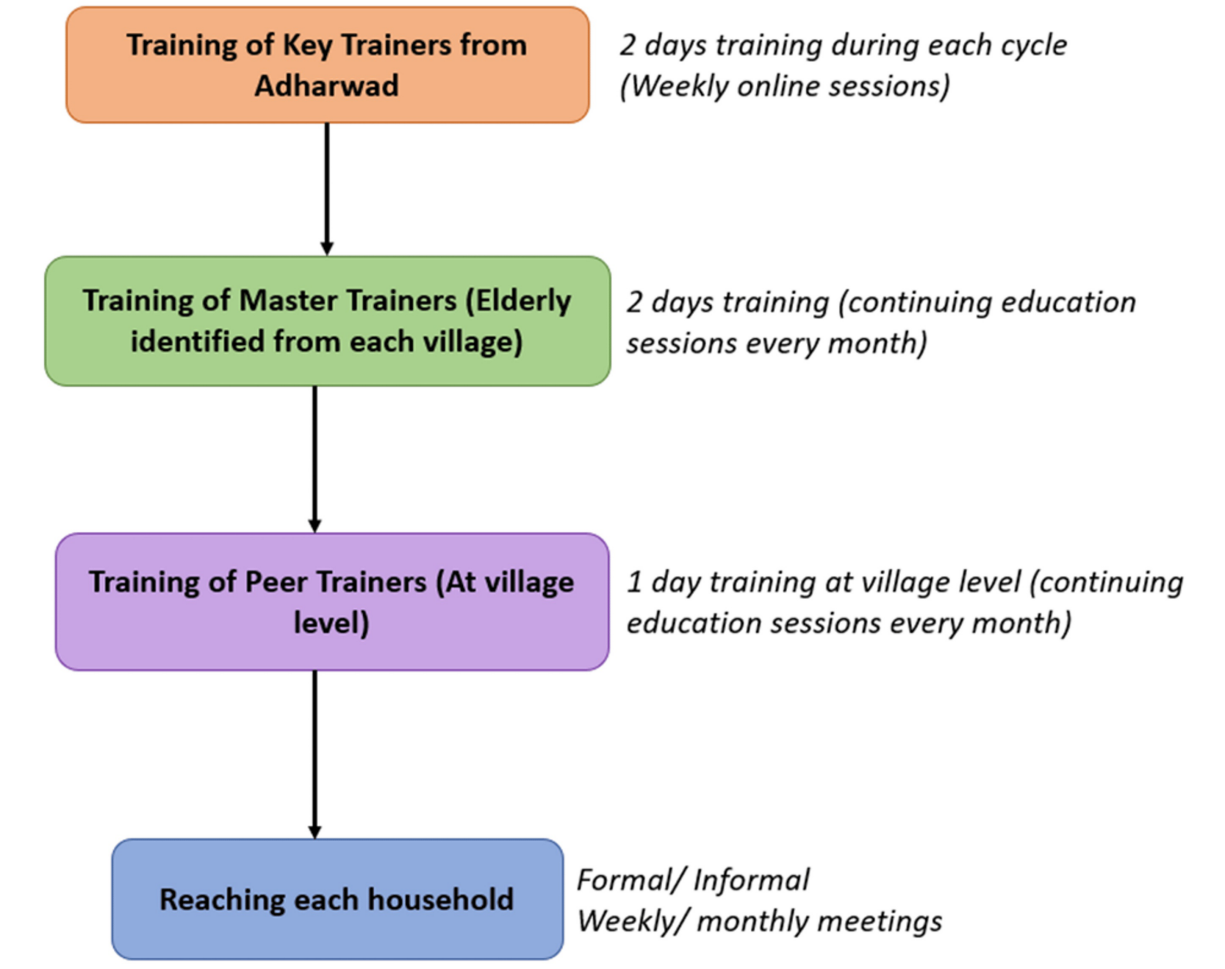
Training flow and cascade model for the intergenerational program Source: Created by the authors using Microsoft PowerPoint (Microsoft Corporation, Redmond, WA, US).

Figure 2 and Table 1 show the cascade training model adopted in the project to ensure widespread community engagement and sustainable knowledge sharing. The process begins with the training of Key Trainers from Adharwad who undergo a two-day training during each cycle, along with weekly online sessions for ongoing learning. These key trainers then conduct the training of Master Trainers, elderly individuals identified from each village, through two-day sessions supplemented by monthly continuing education. The trained master trainers are responsible for training Peer Trainers at the village level in one-day workshops, followed by regular monthly sessions to reinforce the learning. They reach out to community members through formal and informal meetings held weekly or monthly, thus ensuring that key messages and practices related to intergenerational engagement and elderly self-care are disseminated to every household. This structured and layered approach builds local capacity, fosters community ownership, and strengthens intergenerational bonds at the grassroots level.

**Table 1:** Roles and responsibilities of trainers in the intergenerational program Source: Created by the authors.

Group	Role	Responsibilities
Key Trainers/Facilitators	Conduct training for master trainers	Train master facilitators in workshops; selected members from Adharwad
Master Trainers/Facilitators	Conduct workshops for peer facilitators	Two elderly (50+) facilitators selected; the number increased in Phase II
Peer Trainers/Facilitators	Spread messages in the community	Selected older volunteers (50+); expected to spread messages informally or formally at the village level

Three-Component Curriculum

Responsive caregiving for young children: In many rural Indian households, grandparents often serve as secondary caregivers, especially when parents are away for work. The curriculum emphasizes the importance of responsive feeding, play-based learning, emotional bonding, and creating a safe and stimulating home environment. Elders also receive guidance on how to engage with children through storytelling, traditional games, and affectionate communication, reviving cultural practices that support socialization and learning.

Elders’ involvement in adolescent well-being: The program encourages intergenerational dialog, allowing adolescents to benefit from the lived experiences and wisdom of older adults while fostering mutual respect and understanding between age groups. Elders are trained to serve as mentors and guides, helping adolescents navigate challenges related to identity, peer pressure, and future aspirations. The curriculum includes sessions on how elders can support adolescents in developing life skills such as decision-making, emotional regulation, communication, and resilience.

Self-care of the elderly: This part of the curriculum covers essential topics such as nutrition, chronic disease management (e.g., hypertension and diabetes), mental health awareness, physical activity, fall prevention, and the importance of social interaction. Sessions also focus on recognizing danger signs, maintaining hygiene, and accessing health services. Practical tools, such as self-check routines, peer support networks, and community-based wellness activities, are introduced to help elderly participants maintain their independence and quality of life.

Participatory activities: These included focus group discussions and in-depth interviews with elderly participants, frontline workers, and facilitators to understand caregiving roles, skill-sharing, and perceptions of intergenerational relationships. Spider web analysis was used to assess participation levels, peer bonding, and increased community respect for the elderly. Force field analysis conducted in all project villages identified key enabling and inhibiting factors affecting elderly involvement in intergenerational activities. Also, oral history sessions, drawing competitions, and intergenerational fairs (*Melawa*) helped build bridges between generations while reviving community bonds.

Local partnerships: Engagement with *ASHA *workers, *Anganwadi* workers (AWWs), and gram *panchayats *(Local Self-Government at the Village Level) ensured grassroots ownership and sustainability.

Impact and insights

The evaluation followed a mixed-method baseline-endline design across 18 villages. A quantitative assessment was conducted using stratified random sampling and standardized tools such as the WHO-QOL BREF [13], Social Capital Scale [14], and Loyola Generativity Scale [15], complemented by qualitative and participatory evaluation methods. There was a notable increase in elders’ self-perceived usefulness and their willingness to contribute to younger generations. Enhanced caregiving by elders led to improved child development outcomes, while greater involvement with adolescents supported life-skill development. The program also helped strengthen social capital and increased community respect for the elderly. Participants reported deeper emotional bonds, stronger intergenerational understanding, and a renewed sense of purpose for older adults.

Scaling intergenerational impact: to move from pilot to policy

The *Sahjeevan* model demonstrates the feasibility of implementing IGPs within rural community systems when grounded in local culture and implemented through existing community systems. Its success in a low-resource, rural setting reinforces that age-integrated models are not only feasible but also deeply impactful when driven by grassroots engagement and participatory frameworks.

To scale this initiative from a promising pilot to a national strategy, it is essential to integrate IGP into flagship government programs such as the National Programme for Health Care of the Elderly (NPHCE) and the Integrated Child Development Services (ICDS). These platforms already reach millions and offer fertile ground for embedding intergenerational activities. Further, frontline workers like *ASHAs* and *Anganwadi* workers can serve as critical agents of change by delivering intergenerational messages during routine home visits, making the model both cost-effective and sustainable. Schools also present a vital entry point. Introducing oral history sessions and mentorship programs can help bridge the often-overlooked caregiving gap for children aged 5 to 10, fostering early empathy, cultural learning, and mutual respect. Additionally, empowering and recognizing local community volunteers, such as members of *Adharwad*, through structured training and public acknowledgment can strengthen local ownership and sustain momentum.

ELITE project: current implementation context

ELITE (Enhancing Living Experiences of the Elderly) is being implemented across approximately 100 villages in Wardha block with the aim of promoting healthy, active, and community-driven ageing. The initiative is structured around village-level *Sanjeevani* (*Sahjeevan*) groups comprising 12-15 older adults per village, supported by 8-10 trained peer facilitators. A cascade training model has been employed, involving orientation of key trainers (Community Health Officers, ASHA facilitators, and civil-society partners), followed by the development of master and peer trainer cohorts. *Sanjeevani* groups conduct structured village sessions focusing on positive aging and reduction of ageism, physical, mental, and social self-care practices, nutrition guidance, basic cognitive exercises, home safety, intergenerational activity planning, and community engagement events.

Conclusion

Aging with dignity and purpose is a fundamental right, yet one increasingly threatened in rapidly changing societies. Intergenerational programs offer a powerful, low-cost, and culturally rooted strategy to rebuild the social fabric while advancing multiple health goals. Experience from Project Sahjeevan underscores the importance of local partnerships, community ownership, and responsive design in making such programs both feasible and transformative.

Global efforts toward healthy aging and child development would benefit from embracing intergenerational models not just as social projects, but as integrated public health interventions with a wide-reaching impact.
